# Serotonin Syndrome Precipitated by Paxlovid Initiation

**DOI:** 10.7759/cureus.42898

**Published:** 2023-08-03

**Authors:** Nicholas G Nasser, Connor Welsh, Avir Mitra, Matthew Swan

**Affiliations:** 1 Emergency Medicine, Mount Sinai Hospital, New York City, USA; 2 Neurology, Mount Sinai Hospital, New York City, USA

**Keywords:** medication interactions, nirmatrelvir and ritonavir, serotonin syndrome, covid-19, paxlovid

## Abstract

A female in her early 40s taking buspirone, quetiapine, and daily kratom presented to the emergency department two days after starting a course of Paxlovid for a mild COVID-19 infection with diffuse tremors, ocular clonus, diaphoresis, and confusion consistent with serotonin syndrome (SS). The patient was treated with oral lorazepam, and her symptoms significantly improved within one hour without the need for additional dosing. To our knowledge, this is the first reported case of SS in a COVID-19-positive patient who was prescribed Paxlovid. Clinicians should be mindful of the potential interactions of Paxlovid with serotonergic agents, and they should consider reducing the dose of these agents or selecting other therapeutics to treat COVID-19 infection in these patients.

## Introduction

Throughout the COVID-19 pandemic, several antiviral medications have been studied for their effectiveness and safety in combating the novel pathogen SARS-CoV-2. One such antiviral, ritonavir, an HIV-1 protease inhibitor, has previously been shown in case reports to trigger serotonin syndrome (SS) in patients concomitantly taking serotonergic agents [1]. SS is a potentially life-threatening drug-mediated disorder caused by the over-activation of peripheral and central postsynaptic 5-HT_1A_ and 5-HT_2A_ serotonin receptors [2].

Two case studies involving the experimental use of the combination therapy lopinavir-ritonavir (LPV/r) in hospitalized patients with severe COVID-19 have demonstrated the precipitation of SS in the setting of concomitant use of serotonergic agents [3]. Paxlovid, a combination of nirmatrelvir and ritonavir, came to market under emergency use authorization (EUA) in December 2021 as a treatment for patients with mild-to-moderate coronavirus disease who are at high risk for progression to severe disease. To date, there have been no reported cases of patients with COVID-19 taking Paxlovid presenting with SS. 

In this report, we will explore a case of a female in her early 40s prescribed Paxlovid for a confirmed COVID-19 diagnosis who developed SS in the setting of concomitant use of agents whose toxicity likely precipitated this effect.

## Case presentation

A female in her early 40s with a past medical history significant for bipolar II disorder, anxiety, depression, and a recent COVID-19 diagnosis with mild symptoms who was initiated on Paxlovid two days prior presented to the emergency department with diffuse tremors and the inability to ambulate. The patient’s partner noted she had multiple falls and worsening confusion over the past day; there were no episodes of syncope or any noted head strikes. Other pertinent negatives included no fevers, chills, chest pain, headaches, changes in urination or bowel movements, and no recent head trauma.

Home medications for this patient include buspirone 30 mg (one tablet in the morning and two tablets at night), clonazepam 2 mg as needed (typically takes one tablet every three days and no recent changes), lamotrigine 200 mg (two tablets at night), quetiapine 300 mg (one tablet in the evening), zaleplon 10 mg as needed, and kratom (*Mitragyna speciosa*) powder two to three teaspoons daily. The patient denied using any alcohol or other substances.

On arrival at the emergency department, the patient was alert and oriented to person, place, and time; however, she was confused and would frequently repeat questions and answers or ignore them entirely. Her behavior was erratic, at times excitable. Her vitals were within normal limits, with a heart rate of 90 beats per minute, a respiratory rate of 18 breaths per minute, blood pressure of 106/68, and normal temperature. Her exam was significant for diaphoresis, bilateral dilated pupils that were minimally responsive to light stimulation with noted ocular clonus, or involuntary multidirectional oscillatory eye movements. She also was noted to have diffuse tremors that were more pronounced in her upper extremities with no rigidity. Of note, the patient was unable to complete rapid alternating motions or finger-to-nose testing due to upper extremity tremors; the tremors were worse with movements. The patient was able to lift all four extremities against gravity with no focal weakness or sensory deficit. Cranial nerves II-XII were intact, and there was no observed myoclonus. Reflexes were brisk and equal in her bilateral upper and lower extremities; no hyperreflexia was observed.

**Figure 1:**
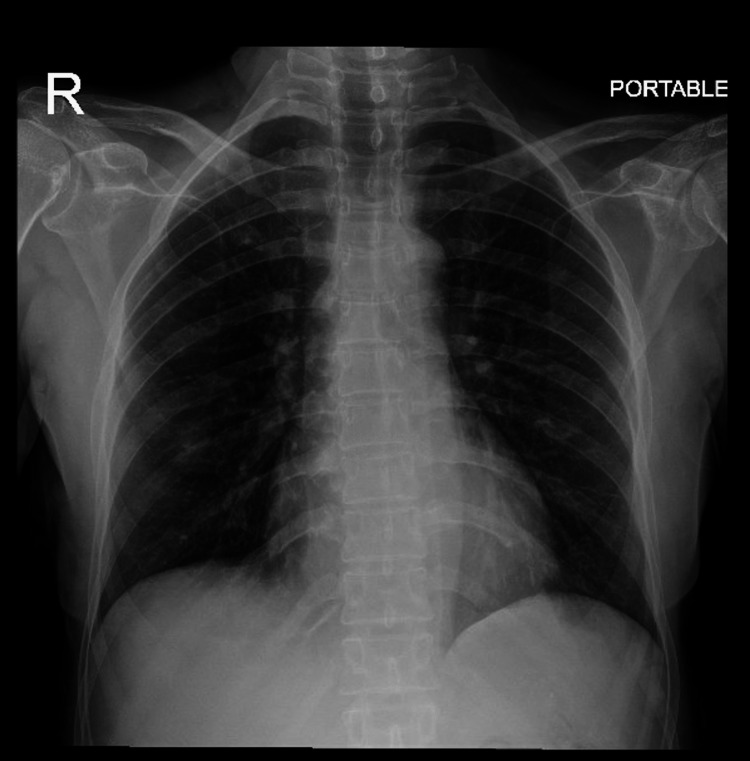
Chest X-ray

**Figure 2:**
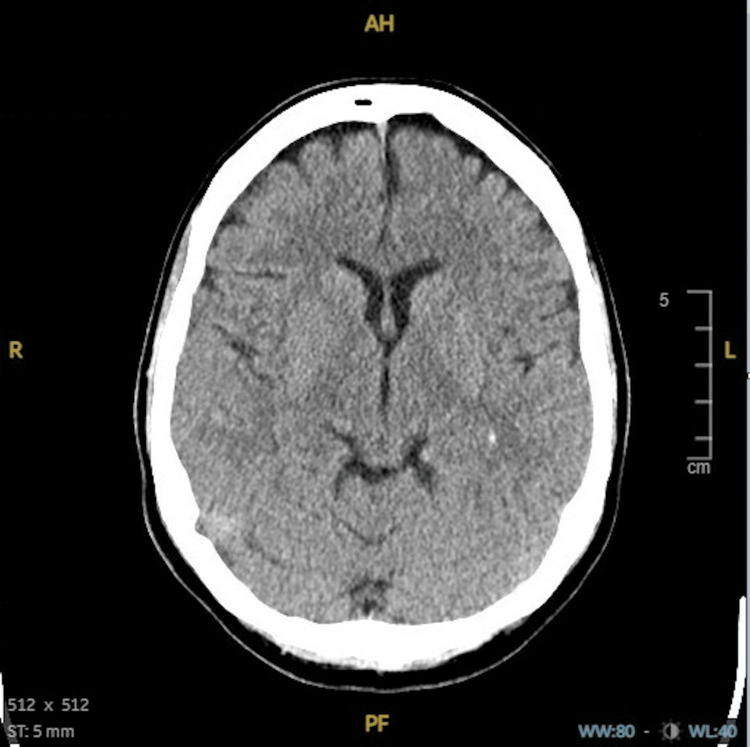
CT head without contrast

Initial studies included a complete blood count, basic metabolic profile, hepatic function profile, magnesium and phosphorus serum levels, creatinine kinase, thyroid-stimulating hormone, venous blood gas, ethanol level, urine pregnancy test, urinalysis, and urine toxin screen, which were all unremarkable. The patient received a chest X-ray and a computed tomography (CT) of her head without contrast, which were also unremarkable (Figures 1, 2). An electrocardiogram demonstrated normal sinus rhythm. A COVID-19 rapid polymerase chain reaction test that was completed in the emergency department was positive.

Based on the patient’s history, physical exam findings, and negative initial laboratory and radiological workup, there was a significant concern for possible serotonergic toxicity secondary to the initiation of Paxlovid in the setting of multiple serotonergic medications onboard, including buspirone, quetiapine, and kratom. Other considerations included benzodiazepine withdrawal; however, this was unlikely given that the patient had no significant reported changes in intake. Consideration was given for intrinsic intracranial pathology; however, the rapid onset, lack of trauma, and negative CT head made this less likely. Metabolic processes such as hyponatremia, infection, or thyrotoxicosis were excluded via ED workup.

Based on the patient’s history and physical exam, the on-call neurology team agreed that the most likely explanation for the patient’s symptoms was SS in the setting of recent Paxlovid initiation. The initial management of the patient’s symptoms included 2 mg of oral lorazepam, and the neurology team was consulted promptly for further evaluation and recommendations. Approximately one hour after benzodiazepine therapy was initiated, the patient experienced a rapid improvement and resolution of her symptoms.

The consulting neurology team recommended discontinuing Paxlovid, continued lorazepam administration as needed, and hospitalization. There was no recommendation for cyproheptadine, given that the patient’s symptoms had improved with lorazepam initially. There was initial consideration for magnetic resonance imaging of the brain with and without contrast to further elucidate any underlying pathology not identified on previous imaging; however, this was not recommended by the consulting team due to the resolution of symptoms and the presumed diagnosis.

The patient was admitted to the internal medicine service and was noted to have only a minimal tremor the following afternoon. The patient was subsequently discharged, given symptom resolution with a diagnosis of SS, and did not require any additional dosing of benzodiazepine therapy. Subsequently, the patient has resumed taking her normal medications at their previous doses and has not experienced any similar symptoms in the six months since her initial emergency department presentation.

## Discussion

As various therapeutics have arrived on the market under EUA throughout the COVID-19 pandemic, providers are continually challenged when it comes to determining the risk of potential medication interactions when prescribing COVID-19 therapies.

SS is a clinically diagnosed dose-dependent cluster of adverse effects caused by increased levels of serotonin in the central and peripheral nervous systems. The Hunter Serotonin Toxicity Criteria is often used as a diagnostic tool, which requires the presence of a serotonergic agent in addition to meeting one of the following criteria: spontaneous clonus, inducible clonus plus agitation or diaphoresis, ocular clonus plus agitation or diaphoresis, tremor plus hyperreflexia, or hypertonia plus temperature above 38°C plus ocular clonus or inducible clonus [4]. In this case, the patient met the criteria for SS based on the use of serotonergic agents (buspirone and quetiapine) in addition to initial presentation with ocular clonus and diaphoresis.

Second-generation antipsychotics, such as quetiapine, have been known to induce SS largely due to antagonism at 5-HT_2A_ receptors, which shunts an increased neurotransmitter effect on other receptors, including 5-HT_1A_ [5]. Likewise, buspirone, a partial agonist of serotonin 5-HT_1A_ receptors, has been documented in several case studies in addition to other serotonergic agents to have contributed to the precipitation of SS [6,7].

Kratom, a loosely regulated herbal medication often purchased in head shops, can provide an opioid-like effect at high doses and is often used in patients with opioid use disorder as a replacement therapy [8]. Several case studies of patients taking serotonergic agents in addition to kratom have developed SS likely due to inhibition of cytochrome P40 enzyme isotypes, including 3A4, 2C9, and 2D6 [9,10]. Thus, in addition to multiple serotonergic agents, our patient was consuming a herbal supplement that had some possible baseline impact on the clearance of these agents. However, this patient reported consuming kratom daily for several months without any dose changes; thus, it is unlikely that kratom was the precipitating factor in this case of SS.

As of December 2021, Paxlovid, a combination drug including the antiviral medications nirmatrelvir and ritonavir, was authorized under the Federal Drug Administration (FDA) EUA for the treatment of mild-to-moderate coronavirus disease in adults and pediatric patients 12 years of age and older who are at high risk for progression to severe disease. Nirmatrelvir functions as a SARS-COV-2 main protease inhibitor, while ritonavir, a known CYP3A inhibitor, has been previously utilized as an HIV-1 protease inhibitor. The EUA-approved dosage for Paxlovid is 300 mg of nirmatrelvir with 100 mg of ritonavir taken together twice daily for five days, with an indication for dose reduction in patients with moderate renal impairment.

According to the FDA Fact Sheet for Healthcare Providers, contraindications for Paxlovid use include co-administration with drugs highly dependent on CYP3A for clearance, specifically for drugs in which elevated concentrations can be associated with morbidity or mortality due to the strong CYP3A inhibitory effect with ritonavir administration. The EUA provides a detailed list of drugs that may be dependent on CYP3A for clearance and others that may result in potentially significant drug interactions, including several antipsychotics, sedatives, and anticonvulsants [11]. Of note, neither quetiapine nor buspirone is absolutely contraindicated with Paxlovid therapy. That said, dose adjustments are recommended for both medications, and it is also important to recognize that polypharmacy with multiple CYP3A substrates can, as in this case, lead to complex multi-drug interactions.

In 2001, a case series involving an HIV outpatient clinic in Atlanta demonstrated several cases of SS in patients taking ritonavir in addition to fluoxetine, a selective serotonin reuptake inhibitor [1]. In addition, LPV/r, which has been widely used experimentally throughout the COVID-19 pandemic, has been shown to induce SS in two patients, one of which was also taking duloxetine and lithium and the other taking risperidone and morphine [3].

## Conclusions

In summary, SS is a rare and potentially lethal disorder characterized by excess serotonergic activity in the setting of serotonergic agent consumption. Paxlovid, a novel therapeutic under EUA containing ritonavir, is a known CYP3A inhibitor and can precipitate toxic effects of other agents dependent on this enzyme for clearance.

To our knowledge, this is the first reported case of SS in a COVID-19-positive patient who was prescribed Paxlovid. We recommend more attention toward the potential drug interactions that can occur with the increased use of Paxlovid during the ongoing COVID-19 pandemic and future outbreaks. Clinicians should be mindful of the possibility of decreased elimination of serotonergic agents with Paxlovid initiation, and consideration should be made toward dose reduction of those agents or selection of other therapeutics to treat COVID-19 infection.
